# Supplementary material for: Assessing the Stability of Molecular Glues with Weighted Ensemble Simulations
Source: J Chem Theory Comput. 2026 Jul 21;22(15):7801–10. doi: 10.1021/acs.jctc.6c00319 (PMC13472130; doi:10.1021/acs.jctc.6c00319)
Supplement: Supplementary file 1 [file ct6c00319_si_001.pdf]

Supporting Information: Assessing the Stability of Molecular Glues with Weighted Ensemble Simulations

Seref Berk Atik[1] and Alex Dickson[1,2]

[1]Department of Computational Mathematics, Science and Engineering, Michigan State University, East Lansing, Michigan 48824, USA
[2]Department of Biochemistry and Molecular Biology, Michigan State University, East Lansing, Michigan 48824, USA

Email: alexrd@msu.edu

Table S1: Residue-level interface contacts between RBM39 and DCAF15 used for the distance metric. All the distance calculations are done between C_α atoms.

RBM39	DCAF15
Asp264	Thr230
Asp264	Phe231
Asp264	Gln232
Met265	Thr230
Arg267	Thr230
Gly268	Pro229
Gly268	Thr230
Gly268	Phe231
Gly268	Ser549
Gly268	Arg552
Ile269	Trp548
Ile269	Ser549
Ile269	Ser550
Ile269	Arg552
Ile269	Lys553
Phe270	Ser549
Glu271	Phe228
Glu271	Ser549
Pro272	Tyr226
Pro272	Pro227
Pro272	Phe228
Pro272	Ser544
Pro272	Gly545
Pro272	Ser546
Pro272	Trp548
Pro272	Ser549
Phe273	Gly545
Leu316	Lys553
Leu316	Ser554
Leu316	Val556
Ala317	Val556

Table S2: Interaction frequencies between glue molecules and proteins (Figure 6).

Atom-Residue Pairs	Frequency
E7820:H5 – DCAF15:Val556	0.221
E7820:H4 – DCAF15:Val556	0.221
E7820:H3 – DCAF15:Val556	0.211
E7820:O1 – DCAF15:Pro233	0.160
E7820:N4 – DCAF15:ARG552	0.129
E7820:H12 – RBM39:ASP264	0.739
E7820:H8 – RBM39:ASP264	0.484
Indisulam:Cl – DCAF15:ARG552	0.542
Indisulam:O1 – DCAF15:PRO233	0.160
Indisulam:H2 – DCAF15:VAL556	0.157
Indisulam:Cl – DCAF15:VAL556	0.134
Indisulam:H10 – DCAF15:ASP264	0.710
Indisulam:H7 – DCAF15:ASP264	0.558
Indisulam:Cl – DCAF15:ILE269	0.122
C4:H2 – DCAF15:VAL556	0.172
C4:O1 – DCAF15:PRO233	0.160
C4:N3 – DCAF15:ARG552	0.151
C4:H10 – DCAF15:PHE231	0.127
C4:H10 – RBM39:ASP264	0.585
C4:H7 – RBM39:ASP264	0.395
C4:H4 – RBM39:ASP264	0.104
C5:H3 – DCAF15:VAL556	0.206
C5:O1 – DCAF15:PRO233	0.165
C5:H11 – DCAF15:PHE231	0.128
C5:H5 – RBM39:ASP264	0.191
C5:H11 – RBM39:ASP264	0.116
C7:H2 – DCAF15:VAL556	0.260
C7:H11 – DCAF15:PHE231	0.183
C7:O1 – DCAF15:PRO233	0.154
C7:H13 – DCAF15:VAL556	0.130
C7:H12 – DCAF15:VAL556	0.124
C7:H14 – DCAF15:VAL556	0.123
C7:H12 – DCAF!5:THR230	0.109
C7:H10 – DCAF15:LEU563	0.108
C7:H4 – RBM39:ASP264	0.124
C7:H12 – RBM39:GLY268	0.118
C7:H14 – RBM39:GLY268	0.118
C7:H13 – RBM39:GLY268	0.113
C7:H11 – RBM39:ASP264	0.109
C10:Cl – DCAF15:ARG552	0.601
C10:H2 – DCAF15:VAL556	0.113
C10:H6 – RBM39:ASP264	0.211
C10:H5 – RBM39:ASP264	0.156
C10:Cl – RBM39:ILE269	0.132

Table S3: Residue-level interface contacts between RBM39 and DCAF15 used for distance calculations in Figure 7.

RBM39	DCAF15
Glu271:CD	Arg178:CZ
Asp264:CG	Arg574:CZ
Ser285:C	Arg594:CZ
Ala317:CA	Asp557:CG
Arg275:CZ	Met170:CA
Arg275:CZ	Met169:CA
Gly268:C	Arg552:CZ
Gln310:CG	Ser546:CB

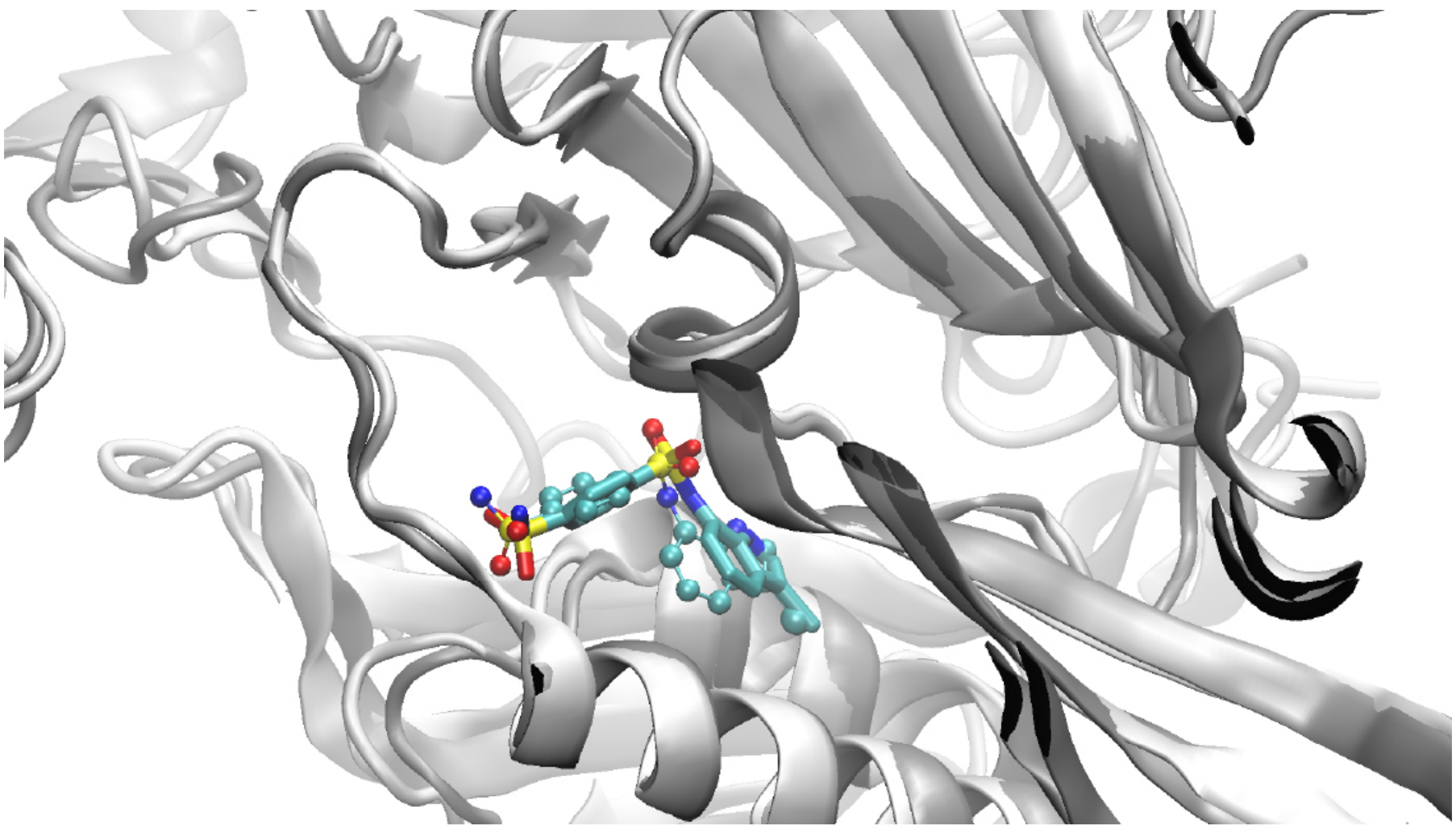

Figure S1: Comparison of the superimposed Indisulam crystal structure (PDB ID: 6SJ7), shown in CPK representation, and the structure generated using the CHARMM-GUI Ligand Reader & Modeler, shown in licorice representation (RMSD = 0.8647 Å).
